# Draft genome sequence of *Phomopsis longicolla* isolate MSPL 10-6

**DOI:** 10.1016/j.gdata.2014.11.007

**Published:** 2014-11-20

**Authors:** Shuxian Li, Omar Darwish, Nadim Alkharouf, Benjamin Matthews, Pingsheng Ji, Leslie L. Domier, Ning Zhang, Burton H. Bluhm

**Affiliations:** a United States Department of Agriculture, Agricultural Research Service (USDA-ARS), Crop Genetics Research Unit, Stoneville, MS 38776, USA; b Department of Computer and Information Sciences, Towson University, MD 21252, USA; c USDA-ARS, Beltsville Agriculture Research Center, Beltsville, MD 21075, USA; d Department of Plant Pathology, University of Georgia, Tifton, GA 31794, USA; e USDA-ARS, Department of Crop Sciences, University of Illinois, Urbana, IL 61801, USA; f Department of Plant Biology Pathology, Rutgers, The State University of New Jersey, New Brunswick, NJ 08901, USA; g Department of Biochemistry and Microbiology, Rutgers, The State University of New Jersey, New Brunswick, NJ 08901, USA; h Department of Plant Pathology, University of Arkansas, Fayetteville, AR 72701, USA

**Keywords:** Draft genome, *Phomopsis longicolla*, Phomopsis seed decay, Sequences, Soybean pathogen

## Abstract

*Phomopsis longicolla* is the primary cause of Phomopsis seed decay in soybean. This disease severely affects soybean seed quality by reducing seed viability and oil content, altering seed composition, and increasing frequencies of moldy and/or split beans. It is one of the most economically important soybean diseases. Here, we report the *de novo* assembled draft genome sequence of the *P. longicolla* isolate MSPL10-6, which was isolated from field-grown soybean seed in Mississippi, USA. This study represents the first reported genome sequence of a seedborne fungal pathogen in the *Diaporthe*–*Phomopsis* complex. The *P. longicolla* genome sequence will enable research into the genetic basis of fungal infection of soybean seed and provide information for the study of soybean–fungal interactions. The genome sequence will also be valuable for molecular genetic marker development, manipulation of pathogenicity-related genes and development of new control strategies for this pathogen.

**Table:** 

Specifications	
Organism/cell line/tissue	*Phomopsis longicolla*
Strain	MSPL 10-6
Sequencer or array type	Illumina HiSeq 2500 sequencer
Data format	Raw and processed
Experimental factors	DNA extracted from a wild-type strain, no treatment
Experimental features	Genome sequencing
Consent	n/a
Sample source location	Soybean field in Stoneville, Mississippi, USA

## Direct link to deposited data

Deposited data can be found here: http://www.ncbi.nlm.nih.gov/nuccore/AYRD00000000.

## Materials and methods

### Pathogen isolation, identification, and pathogenicity test

*Phomopsis longicolla* is the primary cause of Phomopsis seed decay in soybean [1], [2]. An isolate of *P. longicolla*, MSPL10-6, was isolated from field-grown soybean seed in Stoneville, Mississippi, USA in 2010 using the standard seed plating procedure [3]. Briefly, soybean seeds were surface-disinfected in 0.5% sodium hypochlorite for 3 min, rinsed in sterile distilled water, and then placed on acidified potato dextrose agar (APDA) medium (Difico Laboratories, Detroit, MI) that was adjusted to pH 4.8 with 25% lactic acid after autoclaving. Seeds were placed on each Petri dish and incubated at 24 °C for 4–7 days [4].

Putative fungal colonies were selected and examined under a microscope. *P. longicolla* was identified using morphological characteristics according to Hobbs et al. [1]. Further identification was confirmed by the analysis of the ITS region of rDNA amplified by PCR with primers ITS1, 5′-TCCGTAGGTGAACCTGCGG-3′ and ITS4, 5′-TCCTCCGCTTATTGATATGC-3′ [5].

Pathogenicity tests were performed using a cut-seedling inoculation assay [6]. Soybean seed of a susceptible cultivar, Williams 82 was used in the tests. Mycelial plugs (4-mm in diameter) from the margin of a 10-d old culture on APDA were punched out with the large ends of disposable micropipette tips (200 μl). The micropipette tip containing the fungal mycelium was subsequently placed over a 2-wk old cut soybean stem that was cut at just below the first trifoliolate node. Micropipette tips containing plugs of non-infested APDA were served as the negative control. Two days after inoculation, micropipette tips were removed. At 7 days after inoculation, the main stem length was measured from the soil line to the top of the plant, and the lesion on the stem was measured.

### DNA extraction, library construction, and sequencing

Genomic DNA of the *P. longicolla* MSPL10-6 isolate was extracted from a 4-d-old culture using a Qiagen DNeasy Plant Mini Kit (Qiagen Inc., Valencia, CA) and used to generate sequencing libraries. No-gel mate-pair libraries were generated with the Nextera Mate-Pair Sample Preparation kit (Illumina San Diego, CA), and paired-end libraries with the TruSeq DNA PCR-Free Sample Preparation kit (Illumina San Diego, CA) according to the manufacturer's protocols. Libraries were sequenced in separate lanes on an Illumina HiSeq 2500 sequencer using a TruSeq SBS sequencing kit (version 3, Illumina) at the Genomics Core Facility, Purdue University, West Lafayette, IN.

### Data analysis and results

The mate-pair library produced 72,216,734 reads (mean length = 113 bp, insert size = 3,900) with a total of 8.2 billion bp representing 128-fold coverage. The paired-end library produced 63,763,666 reads (mean length = 97 bp, insert size = 524) with a total of 6.2 billion bp representing 97-fold coverage. A draft of the *P. longicolla* genome was assembled from both libraries with SOAPdenovo assembler version 2.04 [7] into 108 scaffolds of 500 bases or larger; among these, the N50 length was 1,039,102 bp, and the largest scaffold contained 6,247,470 bp. The resulting draft genome sequence was estimated to be approximately 62 Mb in size with an overall G + C content of 48.6%. Gene prediction analysis using Augustus version 2.5.5 [8] yielded a total of 15,738 predicted protein-coding regions. Predicted gene models were annotated using blastp against the UniRed100 database, 8868 (56%) of the gene models had significant matches (E value ≤ 1 e^− 5^) to genes in the database. The *P. longicolla* sequencing and assembly statistics were summarized in Table 1.

## Figures and Tables

**Table 1:** *Phomopsis longicolla* sequencing and assembly statistics.

Sequencing statistics library	Raw data		Processed data	
Size	Coverage	Size	Coverage
Paired end 0.5 Kb inserts	6.9 Gb	108 ×	6.2 Gb	97 ×
Mate pair 3.9 Kb inserts	16.2 Gb	253 ×	8.2 Gb	128 ×
Total	23.1 Gb	361 ×	14.4 Gb	225 ×


Assembly statistics	Contigs	Scaffolds
Total assembly size	62 Mb	66.7 Mb
Total assembled sequences	12,329	108
Longest sequence length	215 Kb	6.2 Mb
Average sequence length	5.05 Kb	618 Kb
N90 index	2,900	62
N90 length	3.21 Kb	299 Kb
N50 index	662	17
N50 length	26.3 Kb	1.04 Mb
